# Role of regulatory proteins in HIV-1/HTLV-1 coinfection

**DOI:** 10.1186/1742-4690-11-S1-P122

**Published:** 2014-01-07

**Authors:** Dustin Edwards, Klaus Strebel, Robyn Washington Parks, Cynthia Pise-Masison, Claudio Fenizia, Martina Fiocchi, Genoveffa Franchini

**Affiliations:** 1 Animal Models and Retroviral Vaccines Section, National Cancer Institute, National Institutes of Health, Bethesda, MD, USA; 2 Laboratory of Molecular Microbiology, National Institute of Allergy and Infectious Diseases, National Institutes of Health, Bethesda, MD, USA

## 

In HTLV-1 endemic areas, 5 to 10% of HIV-1-infected individuals are also coinfected with HTLV-1/2. Several studies support the finding that dual infection of HIV-1 and HTLV-2 may confer delayed AIDS progression. In contrast, HTLV-1 has been concluded to have either no effect or to increase progression to AIDS in HIV-1-infected individuals. Although Tax-1 is known to influence HIV-1 replication in vitro, other HTLV-1 proteins likely have a role in retroviral coinfection. Here, we are investigating whether HTLV-1 proteins regulate activation of HIV-1 expression in latently-infected cells. In this study, we used B-cells infected with molecular clones that either express both p12 and p8, p12-only, p8-only, or contain knockouts to orf-I, orf-II, or hbz. The HTLV-1-infected B-cells were cocultured with KK1 T-cells latently-infected with HIV-1. Following coculture, cells and media were assayed for viral expression. Analyses of immunoblot and ELISA data suggest that HTLV-1-infected B-cells that express p8 enhanced activation of latent HIV-1 expression. We are currently examining whether cell-to-cell contact with p8 expressing cells is required to reactivate the latently-infected KK1 cells. Results from this study could have an impact on treatment of patients coinfected with HIV-1 and HTLV-1.

